# Supplementary material for: A fault-intrusion-tolerant system and deadline-aware algorithm for scheduling scientific workflow in the cloud
Source: PeerJ Comput Sci. 2021 Nov 2;7:e747. doi: 10.7717/peerj-cs.747 (PMC8576554; doi:10.7717/peerj-cs.747)
Supplement: Supplemental Information 6 [file peerj-cs-07-747-s006.zip › Myproject1.1/docs/org/workflowsim/clustering/BasicClustering.html]

BasicClustering


JavaScript is disabled on your browser.


- Overview
- Package
- Class
- Use
- Tree
- Deprecated
- Index
- Help

- Prev Class
- Next Class

- Frames
- No Frames

- All Classes

- Summary:
- Nested |
- Field |
- Constr |
- Method

- Detail:
- Field |
- Constr |
- Method


org.workflowsim.clustering

## Class BasicClustering

- java.lang.Object
- - org.workflowsim.clustering.BasicClustering

- All Implemented Interfaces:
  :   ClusteringInterface

  Direct Known Subclasses:
  :   BalancedClustering, BlockClustering, HorizontalClustering, VerticalClustering

  ---

    

  ```
  public class BasicClustering
  extends java.lang.Object
  implements ClusteringInterface
  ```

  The default clustering does no clustering at all, just map a task to a job

  Since:
  :   WorkflowSim Toolkit 1.0

- - ### Constructor Summary

    Constructors

    Constructor and Description

    `BasicClustering()`

    Initialize a BasicClustering object
  - ### Method Summary

    Methods

    | Modifier and Type | Method and Description |
    | --- | --- |
    | `void` | `addClustDelay()` For a clustered job, we should add clustering delay (by default it is zero) |
    | `Task` | `addRoot()` |
    | `protected Job` | `addTasks2Job(java.util.List taskList)` Add a list of task to a new job |
    | `protected Job` | `addTasks2Job(Task task)` Add a task to a new job |
    | `void` | `clean()` Delete the root task |
    | `java.util.List<Job>` | `getJobList()` Gets the job list |
    | `java.util.Map` | `getTask2Job()` Gets the map from task to job |
    | `java.util.List<File>` | `getTaskFiles()` Gets the files |
    | `java.util.List<Task>` | `getTaskList()` Gets the task list |
    | `void` | `run()` The main function of BasicClustering |
    | `void` | `setTaskList(java.util.List<Task> list)` Sets the task list |
    | `protected void` | `updateDependencies()` Update the dependency issues between tasks/jobs |

    - ### Methods inherited from class java.lang.Object

      `clone, equals, finalize, getClass, hashCode, notify, notifyAll, toString, wait, wait, wait`

- - ### Constructor Detail


    - #### BasicClustering

      ```
      public BasicClustering()
      ```

      Initialize a BasicClustering object
  - ### Method Detail


    - #### getTaskFiles

      ```
      public final java.util.List<File> getTaskFiles()
      ```

      Gets the files

      **Specified by:**
      :   `getTaskFiles` in interface `ClusteringInterface`

      Returns:
      :   files


    - #### setTaskList

      ```
      public final void setTaskList(java.util.List<Task> list)
      ```

      Sets the task list

      **Specified by:**
      :   `setTaskList` in interface `ClusteringInterface`

      Parameters:
      :   `list` - task list


    - #### getJobList

      ```
      public final java.util.List<Job> getJobList()
      ```

      Gets the job list

      **Specified by:**
      :   `getJobList` in interface `ClusteringInterface`

      Returns:
      :   job list


    - #### getTaskList

      ```
      public final java.util.List<Task> getTaskList()
      ```

      Gets the task list

      **Specified by:**
      :   `getTaskList` in interface `ClusteringInterface`

      Returns:
      :   task list


    - #### getTask2Job

      ```
      public final java.util.Map getTask2Job()
      ```

      Gets the map from task to job

      Returns:
      :   map


    - #### run

      ```
      public void run()
      ```

      The main function of BasicClustering

      **Specified by:**
      :   `run` in interface `ClusteringInterface`


    - #### addTasks2Job

      ```
      protected final Job addTasks2Job(Task task)
      ```

      Add a task to a new job

      Parameters:
      :   `task` - the task

      Returns:
      :   job the newly created job


    - #### addTasks2Job

      ```
      protected final Job addTasks2Job(java.util.List taskList)
      ```

      Add a list of task to a new job

      Parameters:
      :   `taskList` - the task list

      Returns:
      :   job the newly created job


    - #### addClustDelay

      ```
      public void addClustDelay()
      ```

      For a clustered job, we should add clustering delay (by default it is
      zero)


    - #### updateDependencies

      ```
      protected final void updateDependencies()
      ```

      Update the dependency issues between tasks/jobs


    - #### addRoot

      ```
      public Task addRoot()
      ```


    - #### clean

      ```
      public void clean()
      ```

      Delete the root task


- Overview
- Package
- Class
- Use
- Tree
- Deprecated
- Index
- Help

- Prev Class
- Next Class

- Frames
- No Frames

- All Classes

- Summary:
- Nested |
- Field |
- Constr |
- Method

- Detail:
- Field |
- Constr |
- Method
